# The identification of complex interactions in epidemiology and toxicology: a simulation study of boosted regression trees

**DOI:** 10.1186/1476-069X-13-57

**Published:** 2014-07-04

**Authors:** Erik Lampa, Lars Lind, P Monica Lind, Anna Bornefalk-Hermansson

**Affiliations:** 1 Department of Medical Sciences, Occupational and Environmental Medicine, Uppsala University, 75185 Uppsala Sweden; 2 Department of Medical Sciences, Cardiovascular Epidemiology, Uppsala University, 75185 Uppsala Sweden; 3 Uppsala Clinical Research Center, Uppsala University Hospital, 75185 Uppsala Sweden

**Keywords:** Boosting, Interactions, Toxicology, Epidemiology

## Abstract

**Background:**

There is a need to evaluate complex interaction effects on human health, such as those induced by mixtures of environmental contaminants. The usual approach is to formulate an additive statistical model and check for departures using product terms between the variables of interest. In this paper, we present an approach to search for interaction effects among several variables using boosted regression trees.

**Methods:**

We simulate a continuous outcome from real data on 27 environmental contaminants, some of which are correlated, and test the method’s ability to uncover the simulated interactions. The simulated outcome contains one four-way interaction, one non-linear effect and one interaction between a continuous variable and a binary variable. Four scenarios reflecting different strengths of association are simulated. We illustrate the method using real data.

**Results:**

The method succeeded in identifying the true interactions in all scenarios except where the association was weakest. Some spurious interactions were also found, however. The method was also capable to identify interactions in the real data set.

**Conclusions:**

We conclude that boosted regression trees can be used to uncover complex interaction effects in epidemiological studies.

## Background

The assessment of synergistic and antagonistic effects among chemicals in a mixture has been much debated during the years [1]. If the effect of an exposure (chemical) *A* on a target depends on whether or not another exposure *B* is present and on the level/concentration of *B*, the effect is said to be non-additive (interactive). Interactive effects can be either synergistic, if the combined effect is greater than what could have been expected if *A* was present by itself, or antagonistic, if the combined effect is less than what could have been expected from *A* alone. The assessment of interactive effects is in most fields approached by first defining a null model without interactions from which departures would indicate interactive effects. The definition of the null model differs between epidemiology and toxicology; in the former field, it is based on the additivity of risk differences or as product terms in a statistical model and in the latter they are based on the hypothesized biological mechanisms [2].

There have been a number of null models proposed in toxicology [1], but two frameworks have been used extensively in practice, independent action (IA) and concentration addition (CA) [2,3]. The independent action (IA) model depends on statistical independence between exposures, i.e. each exposure acts independently of the other exposures but they all contribute to the outcome. The joint outcome of exposures is then the probabilistic sum. If the exposures are acting in a similar manner and can be substituted for one another in proportion to their potencies, the concentration addition (CA) model is used as null model. The two different approaches can thus yield different estimates of risk, depending on the mechanistic assumptions. If additivity holds for a combination of *N* exposures, the CA model can be written as

(1) $$\overset{N}{\underset{i=1}{\sum }}\frac{c_{i}}{E_{i}}=1$$

In the above equation, *E*_*i*_ represents the concentration of exposure *i* associated with a certain response and *c*_*i*_ represents the concentration of the *i*^th^ exposure in combination with the other *N*−1 exposures yielding the same response. If the left hand side of equation 1 is less than one, there is a synergistic effect and if the left hand side is greater than one, the effect is antagonistic. It can be shown algebraically that this synergy corresponds to an interaction term with a regression coefficient larger than zero in a regression model whereas the antagonism corresponds to an interaction term with a coefficient less than zero. The additive case, i.e. the coefficient for the interaction term being equal to zero, corresponds to equation 1 [4]. Departures from additivity have been demonstrated in experimental settings [5-9] and in epidemiological studies [10,11]. A recent review [12] highlighted some issues pertinent to the analysis of multi-pollutant mixtures in epidemiological data. The mixtures often consist of several correlated pollutants, the pollutants may interact with each other and there may be non-linear relationships with the outcome.

The assessment of interactions in a statistical model requires that interaction terms are present in the model. These terms are tested along with the main effects and the effects are evaluated. As long as the number of parameters in the model are not many compared to the sample size, the parameters and their standard errors can be estimated. When the number of parameters and possible interactions get large, the sample size might not be sufficient to estimate all parameters. An approach similar to that of genome-wide association studies (GWAS) called environment-wide association study (EWAS) has recently been proposed to screen both genetic and environmental data for candidate interacting factors [13-15]. In a first step, candidate factors are selected based on the strength of their marginal associations. Two-way interactions between the selected candidate factors are tested in a second step in which the false discovery rate (FDR, the expected proportion of false positives to the total number of positives) is estimated using a parametric bootstrap method which involves estimating interaction p-values under the null hypothesis of no interaction.

The nature of the data in epidemiological studies with many measured exposures, with an almost indefinite number of possible sizes and compositions of the interactions makes statistical learning methods, i.e. methods that are tailored to find interesting patterns in data, an attractive approach for identification and prediction of the joint effect. Recent applications of statistical learning methods in toxicology has primarily been used to predict toxicological properties from chemical structures and features. Examples include Support Vector Machines [16,17], random forests and K-nearest neighbor classification [18], neural networks [19] and a combination of different methods [20]. This paper presents the results from the analysis of simulated chemical mixtures using a statistical learning method called gradient boosted regression trees (hereafter called boosted CARTs). We simulated an outcome containing interaction and nonlinear effects under four different scenarios and tested the method’s ability to uncover these effects. We also show an analysis of real data relating environmental contaminants to serum bilirubin levels. Serum bilirubin is one of several markers used clinically in the assessment of liver function. The evaluation of mixture effects on serum bilirubin in humans is highly relevant, since several of the contaminants evaluated are associated with liver toxicity [21]. This method of finding plausible interactions is not limited to the study of mixture effects in toxicology and is therefore relevant in many areas where complex interaction effects are likely to exist.

## Methods

### Classification and regression trees

Classification and Regression Trees (CARTs) [22] are very simple yet powerful. They partition the data into a set of disjoint regions and approximate the outcome with a constant value within these regions. This is accomplished via a series of binary splits in the input variables. The CART is grown in a top-down fashion by first finding the variable and split point that optimizes a statistical criterion, e.g. the residual sum of squares. Within each formed subset the optimal split is determined using the subset of observations passing through the previous split. This is repeated until the number of observations left is too low to be split, typically < 10. A CART consisting of a single split is said to have depth one (*d*=1), a CART with two splits is said to have depth two (*d*=2) and so on. CARTs are thus able to fit complex interactions as each split after the first is conditional on the former split. This means that if a higher order interaction is present, its lower order components are also present. A CART of depth *d* can allow interactions of at most order *d* but usually contains combinations of interactions and nonlinear effects, with the latter being handled via successive splits on the same variable. The fitted CART can then be visually assessed for any interactions and/or nonlinear effects. The ability to automatically handle interactions and nonlinear effects makes CARTs attractive in the study of mixture effects. Further details on CARTs can be found elsewhere [23-25].

CARTs are easily interpretable but have several drawbacks. One drawback is the selection bias towards variables with many possible split points [22]. Another issue is that CARTs are highly variable: a small change in the outcome data can lead to a different CART. Purely additive relationships are poorly approximated by CARTs and much information is lost due to the binary splits of the input variables. Predictions from CARTs are usually somewhat crude and they also tend to overfit the data because of the amount of searching done. The price paid is that stable trees that cross-validate well usually consist of no more than a few terminal nodes and are thus not very discriminating [24].

### Stochastic gradient boosting

In the language of statistical learning, single CARTs are called weak learners because of their poor predictive performance. Stochastic gradient boosting [26] (hereafter called boosting) is a numerical technique created around the idea that many weak learners can be combined into a strong learner with superior predictive performance. The goal is to accurately map a set of explanatory variables **x** to an outcome variable *y* via a function *F*(**x**), which is usually called the target function, estimated by an additive expansion

(2) $$\hat{\;F}(x)=\overset{M}{\underset{m=1}{\sum }}\beta_{m}b\left(x;\gamma_{m}\right)$$

where *M* is the number of weak learners; *β*_*m*_ are the expansion coefficients and *b*(**x**;*γ*_*m*_)are individual weak lerners characterized by the parameters *γ*_*m*_[23]. Accuracy is defined by a loss function *L*(*y*,*F*) which represent the loss in predicting *y* with *F*(**x**). A detailed description of gradient boosting is beyond the scope of this paper but with CARTs as the weak learners the algorithm briefly works as follows 

1. Initialize ${\hat{\;F}}_{0}(\text{x})$ to a constant *α*

2. Randomly sample a fraction *η* from the data without replacement

3. Using *η*, compute the negative gradient of the loss function, *z*_*m*_=−∇*L*, and fit a depth *d* CART, *g*(**x**), predicting *z*_*m*_.

4. Update ${\hat{\;F}}_{m}(\text{x})\leftarrow {\hat{\;F}}_{m-1}(\text{x})+\mathrm{\lambda \rho g}(\text{x})$.

5. Iterate steps 2 through 4*M* times.

In step 4, *ρ* is the step size along the gradient and *λ* is a shrinkage parameter which slows down the learning to reduce overfitting. The parameters *M*, *d* and *λ* can be tuned using the bootstrap or cross-validation, although a value of *d*≃5 is often a reasonable starting point [23]. For squared error loss $L(y,F)=\frac{1}{2}{\left(y-F\right)}^{2}$ the negative gradient is the ordinary residual, so each iteration in the above algorithm fits a CART predicting the residuals from the CART fitted in the previous step. For absolute error loss *L*(*y*,*F*)=|*y*−*F*| the negative gradient is the sign of the residual making it more robust to skewed outcomes than the squared error loss function. Loss functions for binary and multinomial data as well as Poisson and time to event (survival) data are also available [27]. The subsampling in step 2 not only reduces computing time but also usually improves predictive performance [26]. A typical value of *η* is 0.5 meaning that in each step a random sample of half the data is used to grow the CART but *η* can be smaller or larger depending on the sample size. More comprehensive descriptions of boosting are given elsewhere [23,26,28-30].

### Variable importance and interpretation

A single CART is easily interpretable, but this feature is lost in the gradient boosted model, which usually contains hundreds or thousands of trees. The gradient boosted model also does not provide regression coefficients, confidence intervals or p-values for the independent variables, so the difficulty of understanding and evaluating the model is increased. Variable importance and partial dependence plots are two tools that aid interpretation. The measure of variable importance in boosted CARTs is based on the number of times a variable is involved in a split, weighted by the squared improvement of the model as a result of the split. The measure thus incorporates both additive as well as interaction effects.

Graphical visualization of the fitted function as a function of one or more of the explanatory variables provides a comprehensive summary of its dependence on the variables, especially if the function is dominated by additive terms and/or lower-order interactions. The partial dependence of a subset *S* of the explanatory variables can be estimated by

(3) $${\hat{\;F}}_{S}\left(x_{S}\right)=\frac{1}{N}\overset{N}{\underset{i=1}{\sum }}F\left(x_{S},x_{-S(i)}\right)$$

where **x**_−*S*(*i*)_ denotes the data values of the variables not in *S*. ${\hat{\;F}}_{S}(x_{S})$ is the effect of a subset *S* of variables on the outcome after accounting for the average effect of the other variables not in *S*. For boosted CARTs, ${\hat{\;F}}_{S}(x_{S})$ can be calculated from the individual trees without reference to the data which would otherwise be computationally very expensive [29].

### Assessment of interaction effects

The *H* statistic was defined by Friedman & Popescu [31] as a measure of interaction strength. The idea behind it is that if two variables *x*_*j*_ and *x*_*k*_ do not interact with each other, the function *F*_*j* *k*_(*x*_*j*_,*x*_*k*_) can be written as the sum of two functions; one that does not depend on *x*_*k*_ and one that does not depend on *x*_*j*_, i.e. *F*_*j* *k*_(*x*_*j*_,*x*_*k*_)=*F*_*j*_(*x*_*j*_)+*F*_*k*_(*x*_*k*_). The statistic *H*_*j* *k*_ is related to the fraction of variance of *F*_*j* *k*_(*x*_*j*_,*x*_*k*_) not captured by *F*_*j*_(*x*_*j*_)+*F*_*k*_(*x*_*k*_) and ranges from 0 to 1, with larger values indicating stronger interaction effects. For two-way interactions $H_{\text{jk}}^{2}$ is defined as

(4) $$H_{\text{jk}}^{2}=\frac{\sum_{i=1}^{N}{\left[{\hat{\;F}}_{\text{jk}}\left(x_{\text{ij}},x_{\text{ik}}\right)-{\hat{\;F}}_{j}\left(x_{\text{ij}}\right)-{\hat{\;F}}_{k}\left(x_{\text{ik}}\right)\right]}^{2}}{\sum_{i=1}^{N}{\hat{\;F}}_{\text{jk}}^{2}\left(x_{\text{ij}},x_{\text{ik}}\right)}$$

where *i*=1,2,…,*N* is the number of observations in the data. The interaction strength *H*_*j* *k*_ is then calculated as $H_{\text{jk}}=\sqrt{H_{\text{jk}}^{2}}$. The *H* statistic is not restricted to two-way interactions and generalizes to interaction effects of any order. *H* can be used to assess whether a particular variable interacts with any other variable by noting that *F*(**x**)=*F*_*j*_(*x*_*j*_)+*F*_−*j*_(**x**_−*j*_) if variable *x*_*j*_ does not interact with any other variable and by inserting the relevant partial dependencies in 4. *H* is not comparable to the traditional way of assessing interactions via regression coefficients as it is more of a relative measure.

Even if an interaction is absent from *F*(**x**) the sample based estimate of *H* will not necessarily be zero as sampling fluctuations may introduce spurious interactions in $\hat{\;F}(x)$. A parametric bootstrap procedure can be used to generate a null distribution for *H* in which artificial outcome data containing only additive effects is generated according to

(5) $${\tilde{y}}_{i}=F_{A}\left(x_{i}\right)+\left[y_{p(i)}-F_{A}\left(x_{p(i)}\right)\right]$$

In equation 5, *p*(*i*) represents a random permutation of the integers 1,2,…,*N* and *F*_*A*_(**x**) is the closest fit to the target containing no interaction effects. This could be accomplished by restricting the depth of the CARTs to *d*=1. Nonlinear effects are still captured by the sequential nature of the boosting algorithm even if the individual CARTs are restricted to contain a single split. Other methods could also be used to fit the additive model, e.g. using Generalized Additive Models [32]. The full model is then fitted to the data ${\left\{{\tilde{y}}_{i},x_{i}\right\}}_{1}^{N}$, where **x** are the original data. *H* is then calculated and corresponds to what could be expected if no interactions are present in the target function. The process is repeated many times, and a null distribution for *H* is obtained, which is hereafter denoted by *H*^0^. By comparing the observed value of *H* to *H*^0^, an idea is obtained of which variables participate in interactions and the order of these interaction effects [31].

### Simulations

Our simulated data was based on real data from The Prospective Investigation of the Vasculature in Uppsala Seniors (PIVUS) study [33]. PIVUS is a prospective cohort study with the primary aim to evaluate the usefulness of different measurements of endothelial function and other techniques to evaluate vascular function. Eligible for the study were all individuals aged 70 years living in in the community of Uppsala, Sweden in 2001. Individuals were randomly selected from the population registry, and a total of 1,016 individuals participated in the baseline investigation giving a participation rate of 50.1%. The subjects went through an extensive physical examination and were subjected to blood withdrawal. Blood samples were drawn in the morning after an overnight fast. A total of 37 environmental contaminants, representing different classes, were measured in blood. The study was approved by the Ethics Committee of Uppsala University and all the participants gave their informed consent before the study. More details on the cohort can be found elsewhere [34].

Contaminants measured in blood are often right skewed so the contaminants in our simulated data were assumed to follow log-normal distributions with log scale means and standard deviations set to the empirical estimates from the log transformed contaminants in the PIVUS data. This approximation was not perfect but yielded distributions for the simulated contaminants that closely resembled the real contaminant distributions. We allowed the PCBs to correlate to varying degrees, and PCBs 118, 153, 170 and 209 are used to represent a total of 14 PCBs [35], so our simulated dataset consists of 27 contaminants. Sex was simulated as independent Bernoulli random variables with equal probabilities for males and females. We set our sample size to 1,000. The target function *F*(**x**_*S*_) was generated, very much inspired by [31], according to

(6) $$\begin{array}{l} F(x_{S})=11\cdot e^{-3\left(1-s{\left[\text{PCB 170}\right]}^{2}\right)}\cdot e^{-3\left(1-s{\left[\text{p,p’-DDE}\right]}^{2}\right)}\cdot \\ \;e^{-2\left(1-s{\left[\text{MMP}\right]}^{2}\right)}\cdot e^{-2\left(1-s{\left[\text{Cd}\right]}^{2}\right)}\\ \;-1.6\overset{2}{\sin}\left(\pi \cdot s\;\left[\text{OCDD}\right]\right)\\ \;+s\;\left[\text{BPA}\right]\left(0.6+1.8\cdot I[\;\text{Sex}=\text{Male}]\right) \end{array}$$

The function *s*[ *x*] in equation 6 transforms *x* to range somewhat uniformly between 0 and 1 for numerical convenience and *I*[*Ω*] equals 1 if the logical condition *Ω* is true and 0 otherwise. The variables selected in 6 were chosen so that one of the correlated PCBs as well as one contaminant from each class (metals, phthalates) would be part of the target. The target function, *F*(**x**_*S*_), thus includes a four-way interaction between PCB 170, p-p’-DDE, MMP and Cd and a non-linear dependency on OCDD which is U-shaped on the log-scale. We also included a BPA by sex interaction.

The response *y* was then generated as *y*_*i*_=*F*(**x**_*i* *S*_)+*ε*_*i*_ where *ε*_*i*_∼*N*(0,*σ*) with *σ* chosen to obtain signal to noise ratios (SNRs) of 2, 1, 0.5 and 0.1 respectively. The signal to noise ratio is defined as $\text{SNR}=\frac{\sigma_{F(x_{S})}^{2}}{\sigma^{2}}$, i.e. the ratio of the target function’s variance to the noise variance. A large value of the SNR indicates more signal than noise and a stronger relationship between the outcome and the predictors. The SNRs were chosen to represent a strong relationship (SNR = 2), a moderate relationship (SNR = 1), a weaker relationship (SNR = 0.5) and a very weak relationship (SNR = 0.1). The coefficients were chosen so that each variable in equation 6 would have approximately the same relative influence when SNR = 2. The SNRs present in the simulated data were within 10% of the target SNRs.

#### Tuning of model parameters

We chose the squared error loss function in the analyses of the simulated data. We estimated the optimal number of CARTs to include in the ensemble (*M*) as well as the optimal tree depth (*d*) using the bootstrap. The coefficient of determination, *R*^2^, was evaluated over a grid consisting of all combinations of *M*=100,200,…,12,000 and *d*=1,2,…,10 using 250 bootstrap replicates in each grid point. *M* and *d* were then chosen according to the one standard error (SE) rule which states that we should choose the most parsimonious model with performance within one SE of the optimal model [23]. In this case, with *R*^2^ as the performance metric, we chose *M* and *d* so that *R*^2^ was within one SE of the maximum *R*^2^.

#### Assessing interaction effects

Having selected the model parameters we evaluated the total interaction strength for the ten most important variables. We generated 250 artificial datasets according to equation 5 and visually compared the observed *H* statistics with the null distributions to determine which variables are most likely to be involved in interactions. After the interacting variables had been identified we proceeded to assess two-way interactions by repeating the above process. Since CARTs fit interactions by contruction there is a concern for false discoveries, i.e. declaring an interaction significant when it is not. Moreover, there are no formal rules for assessing the significance of an observed *H* relative to *H*^0^. To get a rough estimate of the false discoveries we performed repeated split-sample evaluations of all interactions deemed important from the visual assessment. The sample was first split in half, creating a training set and a validation set of approximately equal sizes. An ensemble of CARTs with the same parameters as that fitted to the full data was then fitted to the training set, and interactions were evaluated in the validation set. This was repeated ten times to obtain stability measures for the interactions.

All analyses were performed using R version 3.0.1 [36]. The gbm package [27] was used to fit the boosted tree models and the caret package [37] was used for tuning the model parameters. All figures were created using the lattice and latticeExtra packages [38,39].

#### Power simulations

Statistical power is a major issue in finding interactions. To illustrate the power of this method, we performed a less complex simulation in parallel with the main simulation study. Additional file 1 contains the power simulations when the true model contains two-way and three-way interactions. We generated a data set $x={\left\{x_{j}\right\}}_{1}^{20}$ with *x*_*j*_∼*N*(0,1) for various sample sizes and generated two outcome variables according to

(7) $$y_{i1}=1+x_{i1}+x_{i2}+\beta_{12}x_{i1}x_{i2}+\varepsilon_{i}$$

and

(8) $$\begin{array}{l} y_{i2}=1+x_{i1}+x_{i2}+x_{i3}\\ \;+\beta_{12}x_{i1}x_{i1}+\beta_{13}x_{i1}x_{i3}+\beta_{23}x_{i2}x_{i3}\\ \;+\beta_{123}x_{i1}x_{i2}x_{i3}+\varepsilon_{i} \end{array}$$

where *i*=1,…,*N* denotes the individual observations and *ε*∼*N*(0,*σ*), with *σ* chosen to give an SNR of one. The coefficients for the main effects of *x*_1_,*x*_2_ and *x*_3_ were kept constant at one. The coefficients for the interaction terms were set to different permutations of {0.25,0.5,1}. In the model based on equation 8 we restricted *β*_12_,*β*_13_ and *β*_23_ to have the same size. An interaction was declared significant if the observed value of *H* was above the 95^th^ percentile of the null distribution. Data generation, parameter tuning and interaction assessment as described above was performed 100 times for each model.

This was contrasted with the usual approach using parametric models with product terms. In this approach, each variable was first screened for marginal associations with the outcome. P-values from this screening step were collected and adjusted so that the FDR would be controled at 10% [40]. To account for the screening step in the subsequent evaluation of the two-way interaction effects we employed a bootstrap procedure according to 

1. Screen *x*_1_ through *x*_20_ for marginal associations with *y* and retain the p-values.

2. Adjust the p-values so that the FDR is controled at 10%.

3. If *β*_1_ and *β*_2_ are significant, fit a model with the product term *x*_1_*x*_2_ and retain *β*_12_, else set *β*_12_=0.

4. Repeat steps 1 through 3*B* times in samples from the data taken with replacement and calculate a 95% confidence interval for *β*_12_ as the 2.5^th^ and the 97.5^th^ percentiles of the obtained distribution for *β*_12_.

If the confidence interval includes zero, we say that the interaction was not significant. The data generation and evaluation of two- and three-way interactions were repeated 100 times with *B*=100. For the assessment of the three-way interaction, an outer bootstrap loop was created to account for the screening of two-way interactions in which the above mentioned bootstrap procedure was repeated in resampled data. If any two of *β*_12_, *β*_13_ and *β*_23_ could be called significant, *β*_123_ was estimated from a model containing the product term *x*_1_*x*_2_*x*_3_ and its constituent two-way components. If none or only one of *β*_12_, *β*_13_ and *β*_23_ could be called significant, *β*_123_ was set to zero. The outer bootstrap was done 100 times.

### The toxmixepi package for R

An R package containing functions to evaluate possible interaction effects in a boosted CART model using the methods and data described in this paper is available online. Included in the package is the simulated data set used in this paper. The functions in the package are provided as they are and comes with no warranty whatsoever. The package can be installed from R using install_github(“eriklampa/toxmixepi”) (requires the devtools package [41]).

## Results

**Figure 1:**
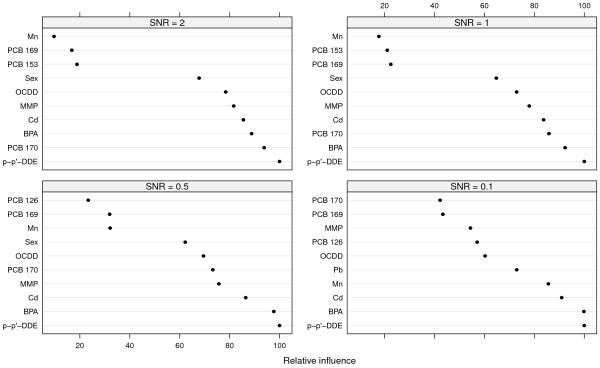
**Variable importance.** Variable importance for the ten most important variables for each SNR. The importance measure has been scaled so that the most important variable has a value of 100.

Table 1 shows the bootstrap validated root mean squared error (RMSE), *R*^2^, the optimal *M* and *d* as well as the *M* and *d* chosen by the one SE rule. Figure 1 shows the ten most influential variables from the different scenarios. The seven variables present in the target function were correctly identified among the ten most important variables in the first three scenarios. For SNR = 0.1, the correct variables were not identified among the top ten as sex came at 14^th^ place in the variable importance ranking. A few unimportant variables (Mn, Pb and two PCBs) placed before PCB 170 in the importance ranking as well.

**Table 1:** **Optimal parameters and parameters chosen according to the one SE rule with** ***R***^**2**^** as the metric**

	**Optimal**				**One SE rule**			
	***d***	***M***	**RMSE**	***R***^**2**^	***d***	***M***	**RMSE**	***R***^**2**^
SNR = 2	8	3,900	1.11	0.57	6	3,500	1.11	0.57
SNR = 1	8	3,000	1.50	0.40	6	2,700	1.52	0.39
SNR = 0.5	10	2,100	2.04	0.23	6	2,400	2.04	0.23
SNR = 0.1	10	1,300	4.29	0.02	5	1,400	4.29	0.02

RMSE and *R*^2^ values are bootstrap validated using 250 resamples.

### Assessment of interaction effects in the simulated data

**Figure 2:**
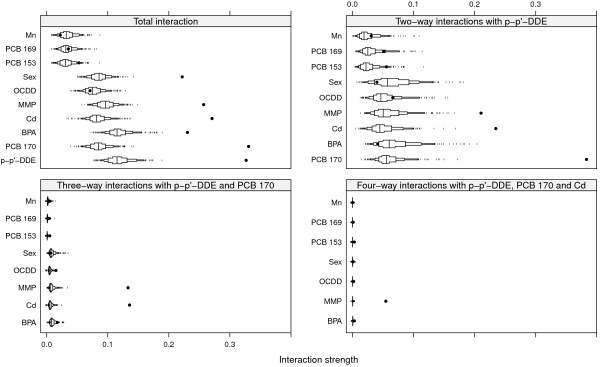
**Interactions for SNR = 2.** Black dots represent observed values of *H*, and boxes represent the null distributions *H*^0^. Small tick marks represent values of the null distribution below or above the 5^*th*^ and 95^*th*^ percentiles respectively.

The top left panel of Figure 2 shows the strengths of the total interaction effects involving each of the ten most influential variables for SNR = 2. Dots are observed values of *H* and boxes represent the derived null distributions of *H* for each variable. We see that p-p’-DDE, PCB 170, BPA, Cd, MMP and sex all seem to be involved in interactions, as the observed values of *H* are well outside the null distribution, whereas OCDD, though it is an important variable, does not seem to interact with any other of the top ten variables.

The top right panel of Figure 2 shows interaction strengths for two-way interactions with p-p’-DDE for SNR = 2. PCB 170 is clearly involved in interactions with p-p’-DDE (stability 10/10), as are Cd (9/10) and MMP (8/10). Sex and BPA were seen to be involved in interactions but do not interact with p-p’-DDE, as their observed values of *H* are well inside their respective null distributions.

The bottom left and right panels of Figure 2 shows interaction strengths for three- and four-way interactions with p-p’-DDE, PCB 170 (left panel) and Cd (right panel) for SNR = 2. The four interacting variables p-p’-DDE, PCB 170, Cd and MMP have been correctly identified as important variables and as variables participating in interactions. These interactions were also identified ten out of ten times in the repeated split-sample validation. The null distributions for *H* in the bottom panels of Figure 2 are very narrow, however, so even small observed values of *H* could become significant.

**Figure 3:**
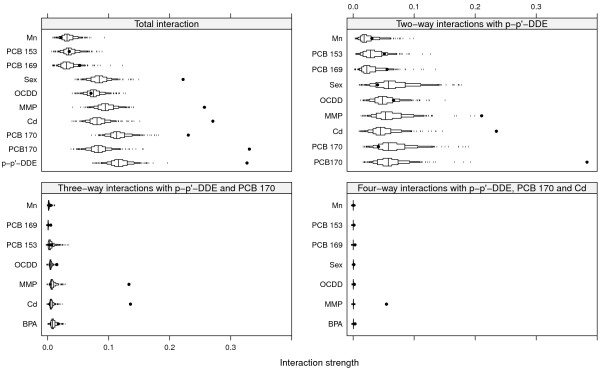
**Interactions for SNR = 1.** Black dots represent observed values of *H*, and boxes represent the derived null distributions *H*^0^. Small tick marks represent values of the null distribution below or above the 5^th^ and 95^th^ percentiles respectively.

**Figure 4:**
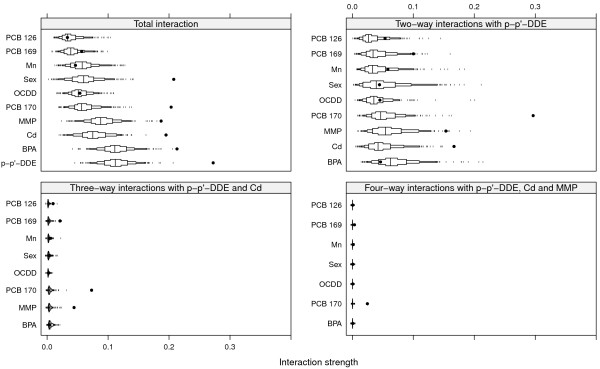
**Interactions for SNR = 0.5.** Black dots represent observed values of *H*, and boxes represent the null distributions *H*^0^. Small tick marks represent values of the null distribution below or above the 5^th^ and 95^th^ percentiles respectively.

**Figure 5:**
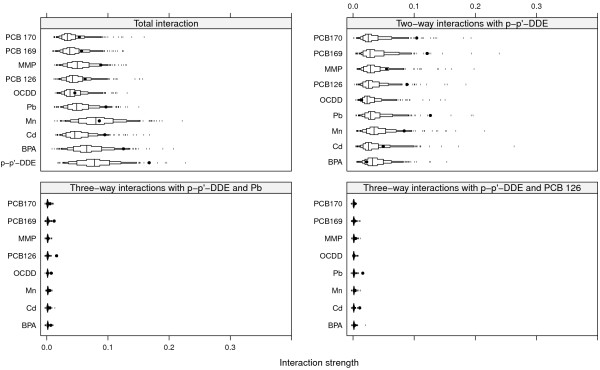
**Interactions for SNR = 0.1.** Black dots represent observed values of *H* and boxes represent the null distributions. Small tick marks represent values of the null distribution below or above the 5^th^ and 95^th^ percentiles respectively.

**Figure 6:**
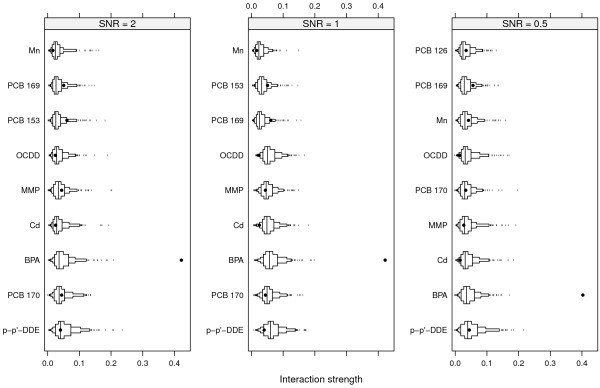
**Two-way interactions with sex.** Black dots represent observed values of *H* and boxes represent the null distributions *H*^0^. Small tick marks represent values of the null distribution below or above the 5^th^ and 95^th^ percentiles respectively.

**Figure 7:**
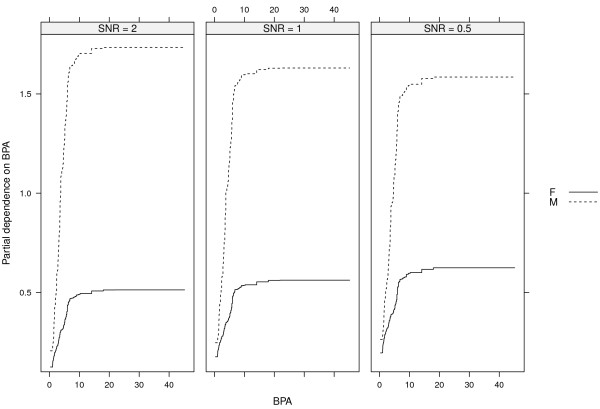
**Partial dependence on BPA.** Partial dependence on BPA conditioned on sex for SNR = 2 (left panel), SNR = 1 (middle panel), SNR = 0.5 (right panel). Solid lines are the partial dependencies for females, and the dashed lines are the partial dependencies for males.

**Figure 8:**
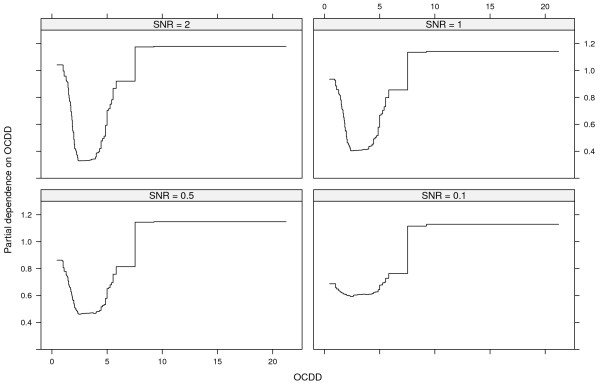
**Partial dependence on OCDD.** Partial dependence on OCDD for SNR = 2 (top left panel), SNR = 1 (top right panel), SNR = 0.5 (bottom left panel) and SNR = 0.1 (bottom right panel).

Figures 3 and 4 show the same for SNR = 1 and SNR = 0.5 as Figure 2 does for SNR = 2. The top left panels of Figures 3 and 4 show the total interaction strengths, and it is clear that the correct interacting variables have been identified. The effect of the narrow null distributions is apparent in the lower left panel of Figure 4. A spurious three-way interactions involving p-p’-DDE, Cd and PCB 169 could be seen, although the observed value of *H* is small. This interaction was less stable (6/10) than the interaction between p-p’-DDE, Cd and PCB 170 (9/10) and between p-p’-DDE, Cd and MMP (9/10) and PCB 169 was not judged to interact with any other variable (Figure 4, top left panel). The correct four-way interactions were identified, however (Figures 3 and 4, lower right panels). The other identified interactions were stable for both SNR = 1 and for SNR = 0.5 (stability ranged between 8/10 and 10/10).The top left panel of Figure 5 shows the strengths of the total interaction effects when SNR = 0.1. Only p-p’-DDE and BPA seem to be involved in interactions and neither the correct two-way interactions (top right panel) nor the correct three-way (bottom panels) interactions were identified. The p-p’-DDE–Pb and p-p’-DDE–PCB 126 interactions were not stable (4/10 and 3/10 respectively) in the split-sample validation and neither was the spurious three-way interaction p-p’-DDE–Pb–PCB 126 (Figure 5 bottom panels, stability 2/10).Figure 6 shows interaction strengths for the two-way interactions with sex for SNR = 2, 1 and 0.5. BPA is clearly interacting with sex in each of the three scenarios (stability 10/10). We did not include SNR = 0.1 in Figure 6 as sex was not found among the ten most important variables. Partial dependences on BPA conditioned on sex are seen in Figure 7 with SNR = 2 (top left panel), SNR = 1, (top right panel) and SNR = 0.5 (bottom left panel). The non-linear dependence on OCDD is captured well as is shown in Figure 8 although the U-shape is not as clear for SNR = 0.1 as it is for the other SNRs.

To summarize, we were able to detect the simulated interactions in all but the noisiest data. Some spurious interactions were also found although they were less stable than the true interactions in the repeated split-sample validation.

### Visualizing the four-way interaction

**Figure 9:**
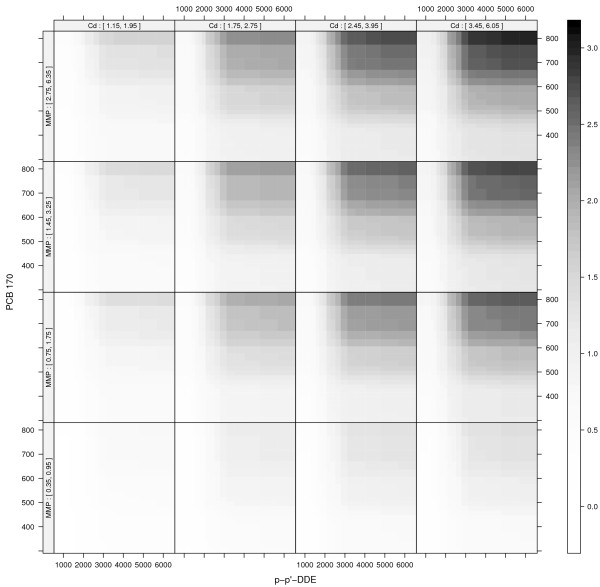
**Visualization of the four-way interaction.** The x- and y-axes of each panel represent p-p’-DDE and PCB 170 respectively. Levels of Cd increase with panels going left to right, and levels of MMP increase with panels going bottom to top. The plotted ranges are from the 10^th^ to the 90^th^ percentiles of each variable’s distribution to ease interpretation.

The four-way interaction between p-p-’DDE, PCB 170, Cd and MMP for SNR = 0.5 is seen in Figure 9. The x- and y-axes of each panel represent p-p’-DDE and PCB 170 levels respectively. Cd and MMP are represented as shingles [38] which are overlapping intervals used to represent continuous variables in a high-dimensional setting. Panels going left to right represent increasing levels of Cd while panels going bottom to top represent increasing levels of MMP. The bar to the right of the figure provides the color codes for the predicted outcome.The bottom left panel of Figure 9 shows the joint effect of p-p’-DDE and PCB 170 while CD and MMP are both at low levels. The synergistic effect is hardly discernable. Following the panels right or up from the bottom left panel shows the joint effect when Cd or MMP increases. The synergistic effect becomes clearer, although it is still small. Following the diagonal from the bottom left panel shows the joint effect of p-p’-DDE and PCB 170 as Cd and MMP both increase, and the synergistic effect is obvious in the top right panel.

### Power simulations

Additional file 1: Figure S1 shows the estimated power to detect a two-way interaction as described above for both boosted CARTs and the parametric model with a product term. Boosted CARTs perform well in comparison with the parametric model except when *β*_12_=0.25 where the parametric model required a smaller sample size than boosted CARTs to achieve >80*%* power. Additional file 1: Figure S2 shows the power to detect a three-way interaction. Here, boosted CARTs outperformed the parametric model when the coefficients for the two-way terms were small.

### Application on real data

In this example we used bilirubin measured in the circulation as the outcome and included 27 environmental contaminants of different classes, sex, education, smoking history, height and weight, medication, blood cholesterol, triglycerides, physical activity and dietary energy intake as predictors. Serum bilirubin levels were transformed using the natural logarithm transformation prior to the analysis. We used the same strategy for tuning the model parameters as for the simulated data.

**Figure 10:**
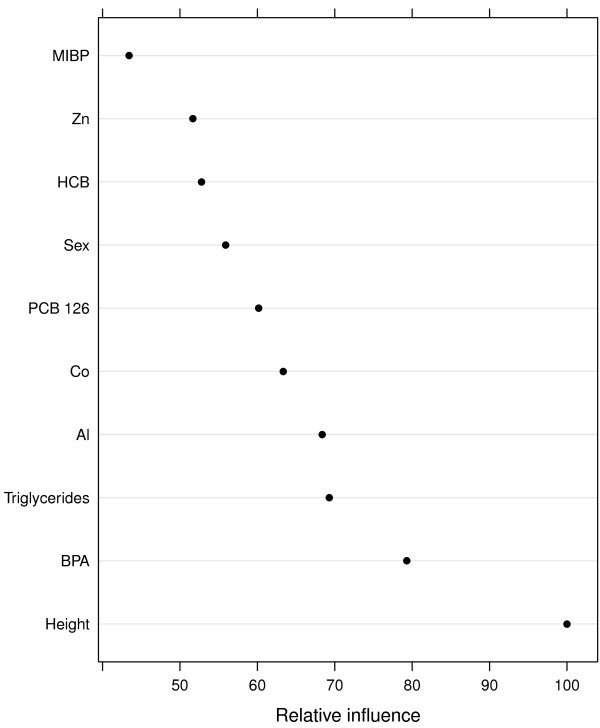
**Variable importance.** The ten most important variables in predicting serum bilirubin levels. The importance measure has been scaled so that the most important variable has a value of 100.

**Figure 11:**
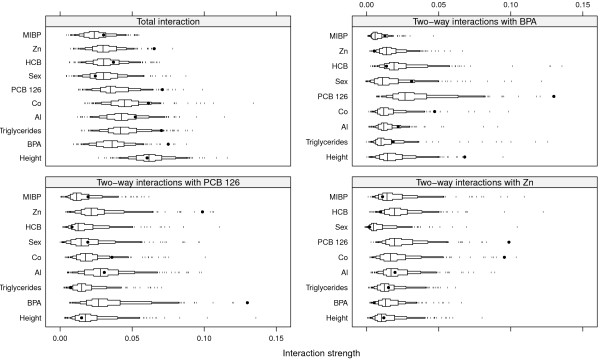
**Interactions.** Black dots represent observed values of *H* and boxes represent the null distributions *H*^0^. Small tick marks represent values of the null distribution below or above the 5^th^ and 95^th^ percentiles respectively.

The maximum bootstrap validated *R*^2^ was 0.19 and was achieved with an ensemble consisting of 6,500 depth 6 CARTs. Using the one SE rule, an ensemble constisting of 6,250 depth 3 CARTs produced a bootstrap validated *R*^2^ of 0.18. The maximum *R*^2^ resulting from an ensemble consisting of CARTs restricted to *d*= 1 was 0.17, suggesting that if interaction effects are present in the data they are not very influential. Figure 10 shows the ten most important predictors of serum bilirubin levels. There were no predictors that clearly stood out from the rest, but height was the most important predictor followed by BPA, Triglycerides, Al and Co. Figure 11 shows the total interaction strength (top left panel), two-way interactions with BPA (top right panel), two-way interactions with PCB 126 (bottom left panel) and two-way interactions with Zn (bottom right panel) for the 10 most important predictors. BPA seems to interact with height (7/10) and PCB 126 (8/10), PCB 126 seems to interact with BPA (7/10) and Zn (8/10) and Zn seems to interact with PCB 126 and Co (stability 7/10 and 2/10 respectively). When assessing the total interaction strength, neither height nor Co seemed to be involved in any interactions (Figure 11, top left panel) and we focus on the interaction involving BPA and PCB 126 in this example.

**Figure 12:**
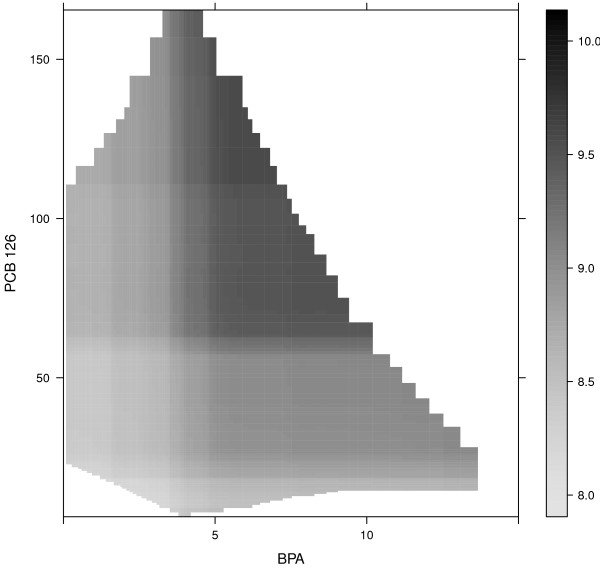
**Joint effect of BPA and PCB 126.** Serum bilirubin levels as a function of BPA and PCB 126. Regions of the joint density of BPA and PCB 126 where there are insufficient data are white.

Figures 12 shows the joint effect of BPA and PCB 126 with darker colors indicating higher bilirubin levles where there are sufficient data as estimated by the perimeter() function in the rms package [42]. Serum bilirubin levels increase with increasing BPA (holding PCB 126 constant at lower levels) and with increasing PCB 126 (holding BPA constant at lower levels). A simultaneous increase in both BPA and PCB 126 further increases serum bilirubin levels suggesting a synergistic effect. Both BPA and PCB 126 have been shown in controlled experiments to be associated with liver toxicity in rats [43,44] so the discovered interaction seems plausible.

## Discussion

In this study, we have shown that boosted regression trees may be a useful tool for uncovering complex interaction effects from a large set of environmental contaminants, as well as non-linear relationships. Simulated data have been used extensively to demonstrate the properties of CARTs, gradient boosting and the *H* statistic [23,28,29,31]. Our study builds on those studies with the addition of correlated variables and different strengths of association. We based our simulated data on real data from the PIVUS study in which the contaminants were measured in the circulation, but the method could as well be applied on other multiple exposure data when complex interactions are likely to exist. Boosted CARTs are very flexible and usually perform very well when faced with the task of predicting a response. Since the search for interactions is fully automatic, the analyst has little control compared to a more traditional approach where subject matter knowledge may dictate where interactions should be sought for. The results from a boosted CART analysis should thus be wieved as exploratory and hypotheses generating until the results have been validated, preferably in external data unrelated to the data used for the analysis. If such external data are not available, the data can be split into a training set and a testing set where the testing set is held out during the modeling process and is only used to test the discovered interactions. Data-splitting is attractive since it allows interactions to be tested in a sample not used in the analysis without requiring external data. However, data-splitting reduces the sample size for both analysis and testing thereby lowering the power to detect interactions. If the sample size is not huge, a different split of the data may lead to different conclusions [24]. The split-sample validation approach suggested here tries to mitigate the last issue, and while not strictly a validation, it may be used as a robustness check as true interactions should be more stable in subsets of data than false ones.

The output from a boosted CART model does not provide confidence intervals or p-values for individual effects as traditional regression methods (i.e. least squares regression, generalized linear models) do. This makes interpretation and understanding of the model more difficult. Partial dependency plots are one way of visualizing the lower-order dependencies. In our example we visualized a four-way interaction using a four-by-four matrix of levelplots. While higher-order interactions are possible to visualize, some information is of course lost, as we cannot graph more than two continuous variables at the same time without resorting to some kind of binning. Confidence intervals for predicted values could possibly be obtained by using the bootstrap. All modeling steps would have to be repeated in each bootstrap sample, and unless *M* and *d* and possibly the shrinkage parameter are fixed from the start, an already resource-intensive method would be even more resource-intensive.

A decomposition of the covariate effects into main and interaction effects is not possible, and we cannot gauge the impact of the interactions as we would in a traditional model. The variable importance measure used for CARTs is based on the number of splits a variable is involved in averaged over the ensemble [23] and captures both additive and interaction effects. We therefore expect to find interactions among only the important variables [31], and the use of the *H* statistics and the derived null distributions can aid in understanding where interactions are most likely to occur. Another option could be to contrast, for each variable, the decrease in mean squared error resulting from splits corresponding to additive effects versus interaction effects. Based on limited simulations, we have seen no obvious advantage over the overall test but it is an approach worth investigating further. Once the interacting variables have been identified and the null distributions simulated, resample based p-values could be calculated as the fraction of *H*^0^ larger than the observed *H*. While this is appealing as it relates to the traditional way of assessing the significance of an interaction term, a potential issue could be the narrow null distributions for higher order interactions. As can be seen in e.g. the lower panels of Figure 4, some null distributions are very narrow and even a small value of *H* could yield a very low p-value and thus be declared significant. Narrow null distributions arise because of how the interaction assessment is done. The null distributions are values of *H* calculated from fits to purely additive data. The numerator in equation 4 will be very small as the joint function will be very similar to the sum of its constituent functions. To interpret interaction effects when the null distributions are very narrow, our recommendation is to create the box-percentile plots so that the x-axis range is common for all investigated interaction orders and visually assess the significance of *H*.

Our proposed method performed well for all but the lowest SNR, which is not surprising considering the relatively small data set and the amount of searching done by the CARTs. The fact that the true interactions were found when SNR was set as low as 0.5 is encouraging and it could be argued, based on these results, that the power to detect interactions is good. The power simulations show that a sample size of 1,000 should be enough to uncover two-way and three-way interactions if the size of the interaction effects are about the same as the main effects and the signal to noise ratio is not low. Naturally, a larger sample size is required to uncover three-way interactions than two-way interactions. Boosted CARTs performed well in comparison with the parametric models with regards to power. The sample size required to achieve >80*%* power for the two-way interactions was larger for boosted CARTs than the parametric model when the coefficients for the two-way product term was small. However, the reverse was observed for the three-way interactions irrespective of the three-way product term’s coefficient. All modeling steps were takien into account for both boosted CARTs and the parametric models and while boosted CARTs may perform worse for the assessment of lower-order interactions for a given sample size, the method’s strength lies in the prediction of higher order interactions as well as nonlinear effects.

Correlated variables are very common in the study of multiple exposures [12]. Despite the correlation between the PCBs in this study, the boosted tree model correctly identified PCB 170 as the most important one in three out of four cases. The correlations between the simulated PCBs were rather low, however, as we used marker PCBs in place of all PCBs measured. We saw some signs of interactions involving PCBs other than PCB 170 (e.g. Figure 4 top right and bottom left panels) and for SNR = 0.1 the wrong PCBs seemed to be involved in interactions. This method thus does not solve the issue with highly correlated exposures and care should be taken when interpreting the results. It is not surprising that *H* is somewhat sensitive to correlated exposures. Nonlinearities are handled in CARTs via successive splits on the same variable and with correlated variables, the CART may choose to split on two or more variables instead of successive splits on one variable, thus creating a spurious interaction. One approach to discourage spurious interactions is to place an incentive for repeated splits on the same variable in the construction of each CART [31]. A very low shrinkage parameter further limits the influence of correlated variables [23]. Correlated exposures could also be summarized into one or more scores using e.g. principal component analysis. While the issues with correlated exposures are solved, the interpretation of the results is much more difficult.

The method outlined in this paper differs somewhat from the EWAS two-step approach in that no screening step is performed. This has the advantage of giving all variables a chance to predict the outcome, and variables with small marginal effects but large interaction effects would end up as relatively influential. The downside is that boosted CARTs are very resource-intensive and it is questionable if this method, in its current state, would be applicable in situations in which data on thousands of genes and environmental factors are measured on many thousand individuals. In situations like those, an EWAS approach [13,14,45] may be a reasonable way to narrow down the list of candidate variables. This screening step would however need to be accounted for in the parameter tuning step.

We used the squared error loss function in our study. This loss function is well suited to situations where the residuals are Gaussian with zero mean and constant variance. In situations where this is not the case, the performance degrades considerably and more robust loss functions should be used. The procedure described in this paper is not limited to continuous outcome variables. For binary outcome variables, which are very common in epidemiological studies, one could generate the artificial outcome data in equation 5 needed for the evaluation of interactions as Bernoulli random variables where the probability of a success is estimated from an ensemble consisting of depth *d*=1 CARTs.

A number of other learning techniques which can accommodate interactions between predictors in a high-dimensional setting merit some attention. RuleFit [46] is an add-on package for R that extracts rules from CARTs and fits them together with linear terms using regularized regression. The framework for detecting interactions presented here was first implemented in RuleFit [31]. At the time of writing, RuleFit can be used to evaluate up to three-way interactions. While interpreting three-way interactions certainly is difficult on its own, it is plausible that higher order interactions may occur in a chemical mixture. Random forests [47] is a technique also based on CARTs. In a random forest, CARTs are grown to full size on bootstrap samples from the data and a random sample of the predictors are used in determining the splits for the individual CARTs. The only tuning parameter in a random forest is the number of predictors to consider for each split and increasing the number of CARTs to add into the ensemble does not lead to overfitting and predictive performance is often comparable to boosting. This makes random forests easy to tune and a very attractive alternative to boosted CARTs. At the time of writing however, functions to extract the necessary partial dependencies needed for the *H* statistic are not implemented for random forests. Multivariate Additive Regression Splines (MARS) [48] fits an expansion of linear basis functions to the data. MARS approximates additive relationships better than CARTs and has the ability to separate main effects from the interaction effects. The method is however less well suited to approximate higher-order interactions [23]. Logic regression [49] share some similarities with CARTs in that they both generate rules, or logical conditions, and was developed to examine interactions in genetic association studies. The main drawback for the type of problems examined here is that Logic regression requires binary predictors. It could be argued that the predictors could be converted to binary form via dichotomization, but that would lead to an unnecessary loss of information. Although CARTs perform binary splits in the predictors, the trees in the ensemble combine to mitigate the problems with dichotomization. Chi-squared automatic interaction detection (CHAID) [50] uses multiple Bonferroni adjusted *χ*^2^-tests and multi-way splits to build prediction rules. The predictors and the response are assumed to be categorical so the same issues regarding continuous predictors as Logic regression applies here. Two relatively new approaches based on the lasso are hierNet [51] and GLINTERNET[52] which try to find two-way interactions subject to hierarchical constraints. Simulations suggest that both hierNet and GLINTERNET outperformed boosting with respect to the FDR [52] although the interaction assessment for boosting was not based on the *H* statistic.

### Suggested workflow

**Figure 13:**
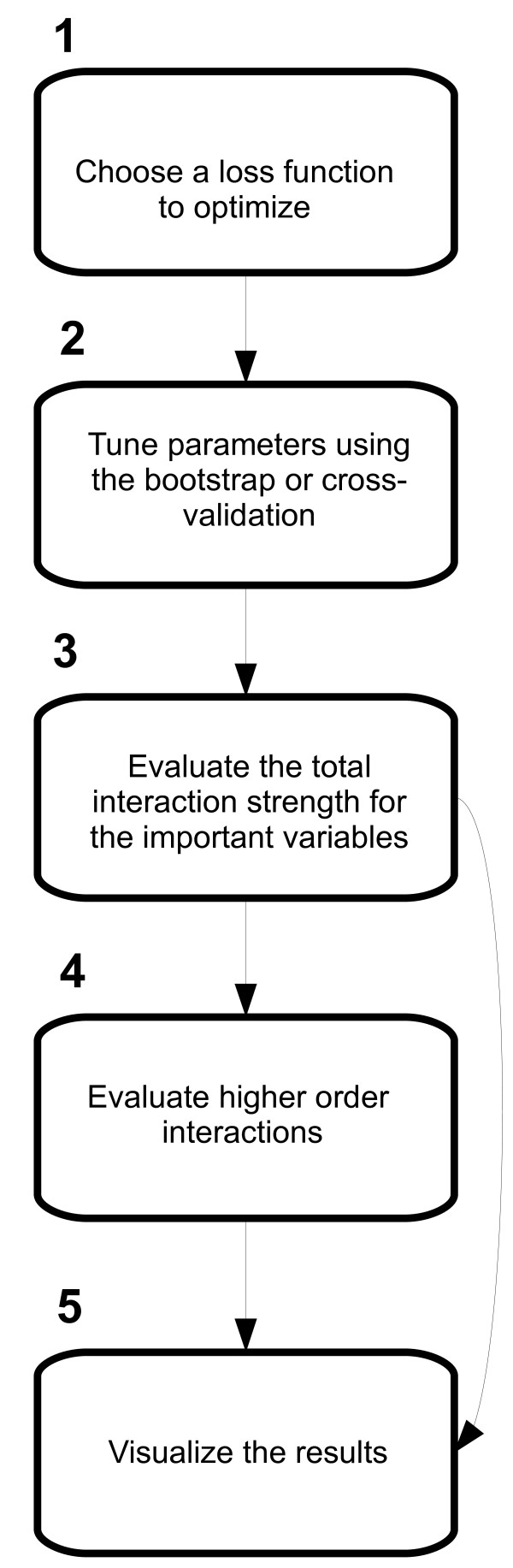
**Suggested workflow.** Conceptual figure showing the suggested workflow of a typical analysis.

Figure 13 shows the workflow for a typical analysis 

1. *Choose a loss function to optimize*. This step is equivalent to choosing the link function in a generalized linear model, e.g. the squared error loss function is similar to ordinary least squares regression and the bernoulli loss function is similar to logistic regression. If the appropriateness of the squared-error loss function is in doubt, the laplace loss function offers a more robust alternative.

2. *Tune the parameters*. Boosted CARTs have three parameters to tune; tree depth, the number of CARTs to include in the ensemble and the shrinkage parameter. The values can be determined by evaluating the performance over a grid of tuning parameter values using the boostrap or cross-validation. We recommend using the one standard error rule when choosing the tuning parameters.

3. *Evaluate total interaction strength*. If *d*>1, there may be one or more interactions present. The total interaction strength can be evaluated for the most important varaibles which is often a smaller subset of all variables included in the analysis. If there is no evidence of interactions, go to step 5.

4. *Evaluate higher order interactions*. When the interacting variables have been identified, the next step is to assess the higher order interactions.

5. *Visualize the results*. Levelplots and/or contour plots can be used to visualize interactions. Additive effects can be visualized using plots of the estimated step functions.

## Conclusions

Boosted CARTs can be used to uncover complex interaction effects and generate hypotheses in epidemiological studies. In this example, simulated as well as real data on environmental contaminants were used to illustrate such interaction effects, but the method could well be applied to other kinds of exposure data.

## Abbreviations

PCB 118: 2,3’,4,4’,5-Pentachlorobiphenyl; PCB 126: 3,3’,4,4’,5-Pentachlorobiphenyl; PCB 153: 2,2’,4,4’,5,5’-Hexachlorobiphenyl; PCB 169: 3,3’,4,4’,5,5’-Hexachlorobiphenyl; PCB 170: 2,2’,3,3’,4,4’,5-Heptachlorobiphenyl; PCB 209: Decachlorobiphenyl; OCDD: Octachlorodibenzo-*p*-dioxin; HCB: Hexachlorobenzene; TNC: *trans*-nonachlor; p-p’-DDE: 1,1-bis-(4-chlorophenyl)-2,2-dichloroethene; BDE47: 2,2’,4,4’-tetra-bromodiphenyl ether; BPA: Bisphenol A; MEHP: Mono-2-ethylhexyl phthalate; MEP: Monoetyl phthalate; MIBP: Monoisobutyl phthalate; MMP: Monomethyl phthalate; Al: Aluminum; Cd: Cadmium; Co: Cobalt; Cr: Chromium; Cu: Copper; Hg: Mercury; Mn: Manganese; Mo: Molybdenum; Ni: Nickel; Pb: Lead; Zn: Zinc; FDR: False discovery rate; SNR: Signal to noise ratio.

## Competing interests

The authors declare that they have no competing interests.

## Authors’ contributions

EL designed the simulations, did the statistical programming and wrote the first draft of the manuscript. ABH contributed to the design of the simulations and helped draft the manuscript. LL and ML participated in the design and coordination of the pivus study and helped draft the manuscript. All authors have read and approved the final manuscript.

## Supplementary Material

Additional file 1 **Power simulations.** Contains the power simulation as described in the text. Click here for file
