# Supplementary material for: The identification of complex interactions in epidemiology and toxicology: a simulation study of boosted regression trees
Source: Environ Health. 2014 Jul 4;13:57. doi: 10.1186/1476-069X-13-57 (PMC4120739; doi:10.1186/1476-069X-13-57)
Supplement: Additional file 1 — Power simulations. Contains the power simulation as described in the text. [file 1476-069X-13-57-S1.pdf]

Power simulations

1 Two-way interactions

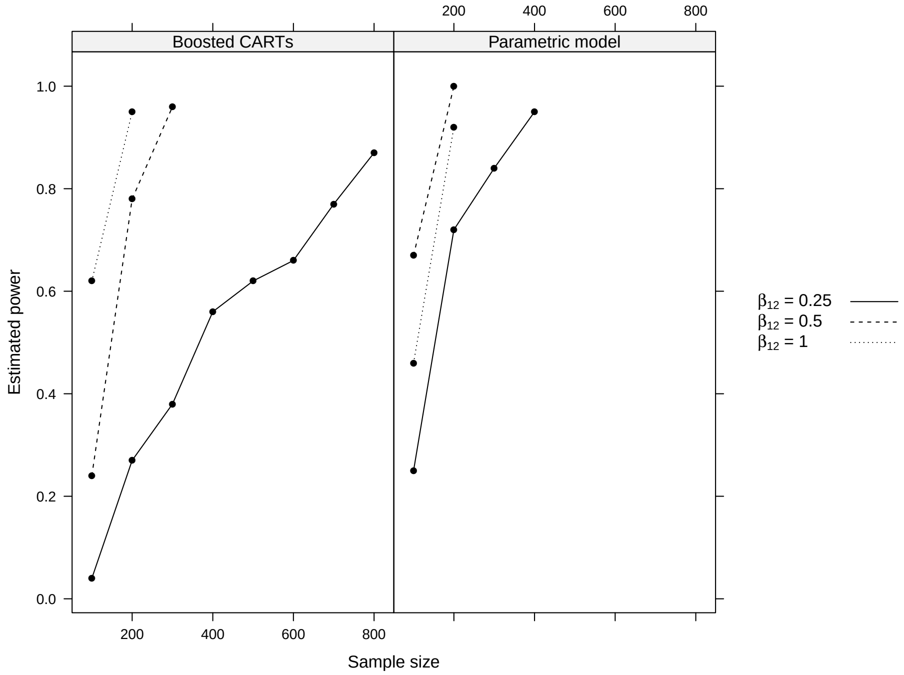

Figure S1: Power to detect the true two-way interaction as a function of the sample size for boosted CARTs (left panel) and a parametric model with a multiplicative term (right panel). The interaction coefficients were set to 0.25 (solid lines), 0.5 (dashed lines) and 1 (dotted lines)

2 Three-way interactions

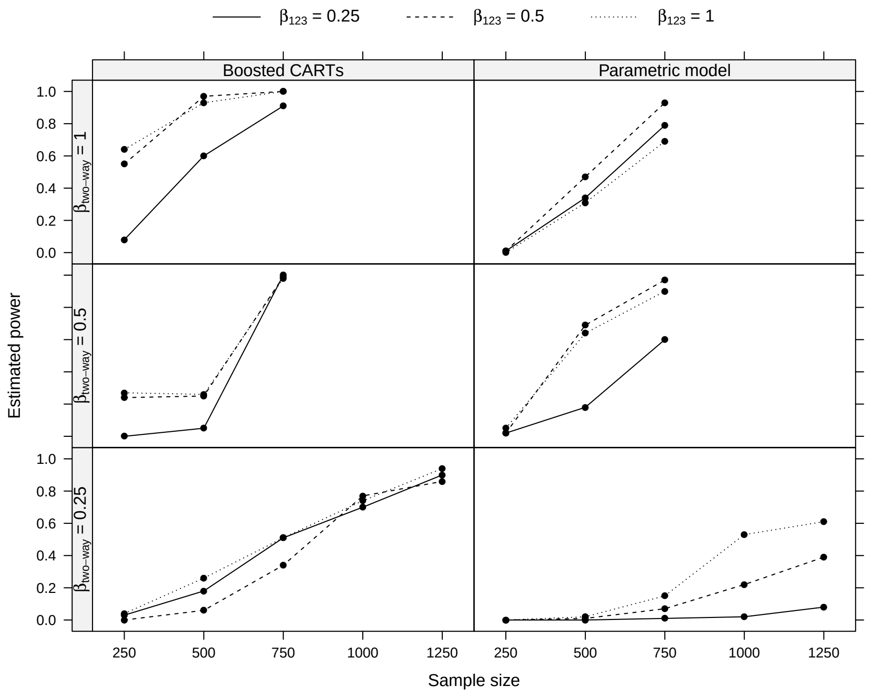

Figure S2: Power to detect the true three-way interaction as a function of the strength of the two-way interaction and the sample size for boosted CARTs (left panels) and parametric models (right panels). The two-way interaction coefficients were all set to 0.25 (bottom panels), 0.5 (middle panels) and 1 (top panels). The three-way interaction coefficients were set to 0.25 (solid lines), 0.5 (dashed lines) and 1 (dotted lines).
